# Detection of Low Humidity Using Three-Dimensional DMC Network Structure

**DOI:** 10.3390/s26051596

**Published:** 2026-03-04

**Authors:** Lu Yang, Xiaomin Chen, Haotian Fan, Huadan Zheng, Jianhui Yu, Wenguo Zhu, Yongchun Zhong, Zhe Chen

**Affiliations:** 1 Key Laboratory of Optoelectronic Information and Sensing Technologies of Guangdong Higher Education Institutes, College of Physics & Optoelectronic Engineering, Jinan University, Guangzhou 510632, China; ylu0208@163.com (L.Y.); chenxm@stu2024.jnu.edu.cn (X.C.); zhenghuadan@jnu.edu.cn (H.Z.); jianhuiyu@jnu.edu.cn (J.Y.); zhuwg88@163.com (W.Z.); thzhechen@jnu.edu.cn (Z.C.); 2 Ji Hua Laboratory, Foshan 528200, China

**Keywords:** low humidity sensing, the DMC/SiO_2_ composite microspheres, three-dimensional network structure

## Abstract

The detection of low humidity levels remains a great challenge in relative humidity (RH) sensing technologies. In this work, methacryloxyethyl trimethyl ammonium chloride (DMC) was coated around SiO_2_ microspheres to form DMC/SiO_2_ composite microspheres, which were self-assembled into a three-dimensional (3D) network structure for low humidity detection. The hydrophilic nature of the DMC component enhances the adsorption capacity for water molecules even at ultra-low humidity levels (1–18.6% RH), while the 3D network structure provides abundant channels for fast water molecule transport, facilitating rapid response and recovery processes. The optimized sensor shows high response (13,544%) in 1–18.6% RH, with short response/recovery time (6 s/10 s) and a small humidity hysteresis (1.4% RH). Such high performance shows that this type of sensor has great potential for application in widespread fields, such as electricity, semiconductor manufacturing, pure gas supply, aerospace, and pharmaceutical formulations.

## 1. Introduction

Humidity is a natural phenomenon resulting from the presence of water vapor in the atmosphere [1,2,3]. Numerous studies have been carried out on sensing technologies for a wide relative humidity range (10–90% RH), due to the relatively low accuracy requirements for sensors applied in general humidity environments [4,5,6,7,8,9]. However, low-humidity sensing is of paramount importance in critical fields [10,11,12], including electricity, semiconductor manufacturing, pure gas supply, aerospace, and pharmaceutical formulations.

For instance, in lithium-ion battery manufacturing, the direct current resistance of battery packs stored in moisture contents higher than 198 ppm (about 0.1% RH at 60 °C) is significantly improved [13]; in solution-processed oxide semiconductor devices, film quality and device performance are highly sensitive to humidity during processing, often requiring conditions below 10% RH [14]; in high-voltage SF_6_ switchgear, moisture must be kept below 800 ppm (about 2.56% RH at 25 °C) to prevent corrosive by-product formation and ensure operational safety [15]. Nevertheless, achieving accurate detection in the low-humidity range (below 10% RH) remains a great challenge in current sensing technology.

In previous studies, various humidity sensitive materials have been used to detect low humidity. For single-component materials, two dimensional materials such as black phosphorus have been used to achieve a rapid 3.9 s response with a sensitivity of 0.2 pF/%RH [16]. Polyelectrolytes leverage ionic dissociation mechanisms to achieve 4800% response within 5–35% RH [17]. Metal halides like NiBr_2_ attain an ultra-high sensitivity of 40 MΩ/%RH via structural transitions but exhibit a long recovery time of 71 s [18,19].

To achieve superior sensing performance, composite materials with different sensitization mechanisms have been employed to attain higher sensitivity at low humidity. Semiconductor-dominated composite materials are centered on metal oxide semiconductors, optimizing charge transport pathways by combining them with conductive materials. For instance, the SnO_2_/MoS_2_ nanocomposite achieves a sensitivity of 387.5 μF/%RH in the 0–20% RH range [20]. The PPy/Ag/TiO_2_ NPs composite exhibits a response of 0.0069 Hz/ppmv in the 173.9–9711 ppmv humidity range with a response time of 12 s [21]. Relying on the ionic conductivity changes in polyelectrolytes, the QC-P4VP/RGO (a polyelectrolyte/polymer-dominated composite) can detect humidity as low as 0.18% RH, providing a new route for ultra-low humidity detection [22]. Porous scaffold-supported composites utilize the synergy of porous structures’ high adsorption and functionalized ions. The SiO_2_/PILs achieves ultra-high 4112% response (7–33% RH) via dual-channel water transport [23,24]. The IL/MOF-AA shows 88% response in the range 5–30% RH, with a 0.6/1.7 s response/recovery time [25]. Despite significant progress, prevailing systems still struggle to simultaneously optimize core metrics, including high sensitivity in ultra-low humidity, rapid response-recovery dynamics, and hysteresis minimization.

In this paper, low humidity sensors based on DMC/SiO_2_ composite microspheres were successfully fabricated. The humidity-sensitive DMC films were uniformly coated around the SiO_2_ microspheres, which self-assembled into a three-dimensional network structure. This integrated design constructs a multi-layered self-supporting 3D DMC-sensitive film network using SiO_2_ microspheres. It effectively prevents the invalid agglomeration of sensitive materials while increasing their effective surface area. Furthermore, the combination of fast molecular transport channels (from the 3D structure) and DMC-sensitive films effectively enhances the water molecule capture efficiency. The obtained humidity sensors exhibit high sensitivity in the low humidity range, ultra-low humidity hysteresis, and rapid response/recovery speed. Due to its excellent performance, this type of humidity sensor will have a wide range of applications in chemical processing, medical diagnostics, electricity, aerospace, and so on.

## 2. Device Fabrication and Characterization

**Figure 1:**
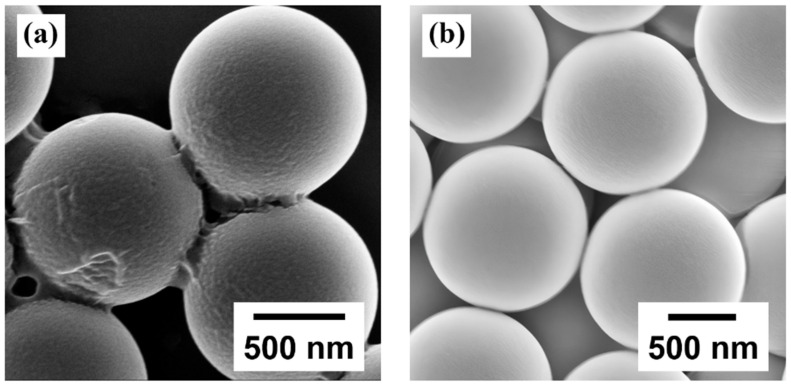
SEM image of (**a**) DMC/SiO_2_ composite microspheres, and (**b**) pure SiO_2_ microspheres.

The DMC/SiO_2_ composite microspheres were synthesized via the electrostatic assembly method at varying DMC-to-SiO_2_ mass feed ratios. Take the composite with a mass feed ratio of 4.4:1 as an example, the fabrication process involves dispersing 0.5 mL of aqueous silica microsphere dispersion (1 µm diameter, 25 mg/mL; Shanghai Yiyuan Biotechnology Co., Ltd., Shanghai, China) in 6.5 mL of deionized water, followed by stirring at room temperature for 5 min and bath sonication for 10 min. Subsequently, 74 mg of DMC solution were added into the prepared suspension. The mixture was stirred for 10 min and ultrasonicated for 10 min. The mixture was maintained at 75 °C in a water bath for 1.5 h. Upon reaction completion, the final product (DMC/SiO_2_ composite microspheres) was collected by centrifugation (4000 rpm, 10 min) and dried in the oven at 40 °C. In our experiment, four different mass feed ratios DMC/SiO_2_ composite microspheres were synthesized (4.4:1, 3.5:1, 2.5:1 and 1.3:1 DMC-to-SiO_2_). Figure 1 presents the scanning electron microscopy (SEM, Ultra-55, Carl Zeiss AG, Oberkochen, Germany) images of the DMC/SiO_2_ composite microspheres and the pure SiO_2_ microspheres. Compared with the pure SiO_2_ microspheres shown in Figure 1b, the DMC/SiO_2_ composite microspheres (Figure 1a) exhibit a slightly rougher surface, indicating that the DMC film forms a uniform coating on the surfaces of the SiO_2_ microspheres.

**Figure 2:**
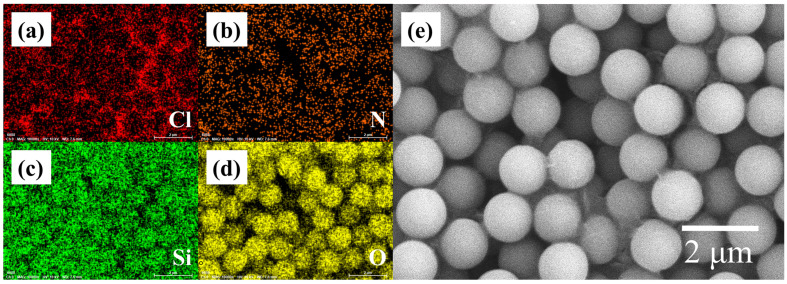
(**a**–**d**) the elemental distribution of Cl, N, Si, and O corresponding to the area in (**e**); (**e**) SEM image of the DMC-coated SiO_2_ composite microspheres.

To further confirm the successful coating of the DMC film on the SiO_2_ microspheres, Energy Dispersive Spectroscopy (EDS) analysis was performed, and the results are shown in Figure 2a–e. Specifically, Figure 2e provides an SEM image of the DMC-coated SiO_2_ composite microspheres, while Figure 2a–d respectively display the elemental distribution of chlorine (Cl), nitrogen (N), silicon (Si), and oxygen (O) corresponding to the area in Figure 2e. Notably, the coating layer on the microspheres contains Cl and N elements (key components of DMC) confirming that the SiO_2_ microspheres are uniformly coated with a DMC film.

**Figure 3:**
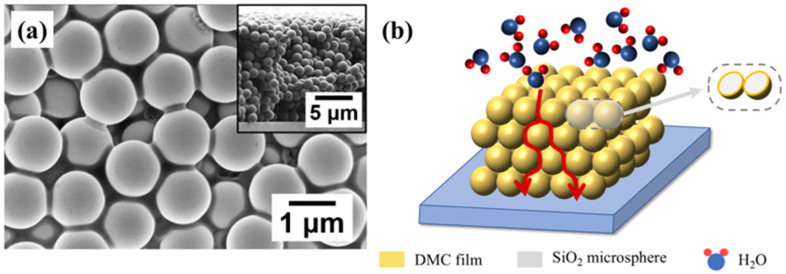
(**a**) SEM image of the 3D interconnected structure assembled from DMC/SiO_2_ composite microspheres, the inset was the cross section of the 3D structure. (**b**) The schematic of the structure of DMC/SiO_2_ sensitive film. The red arrow represents the transport pathway of water molecules.

The resultant microspheres were dispersed in deionized water by sonication with a concentration of 50 mg/mL. Subsequently, 4.0 μL aliquot was drop-casted onto the Si substrate with 60 pairs gold interdigitated electrode (IDE, spacing: 10 μm, finger length: 1.5 mm). After 6 h of natural evaporation at room temperature, the DMC/SiO_2_ composite microspheres were self-assembled into a 3D network structure on the IDEs. As shown in Figure 3a, the DMC/SiO_2_ composite microspheres were arranged into a 3D structure, the right insert of Figure 3a illustrates the cross-section of the 3D structure, which comprises approximately 11 layers and has a total thickness of about 12 μm. As shown in Figure 3b, the SiO_2_ microspheres serve as a supporting scaffold, which effectively prevents the collapse of DMC films. Notably, DMC-sensitive layers (yellow region) encapsulated around the SiO_2_ microspheres (gray region) form a 3D resistive network. Compared to conventional 2D single-layer DMC films, this 3D resistive network offers an enhanced active surface area per unit footprint, thereby maximizing the contact interface between water molecules and the DMC-sensitive films to boost the sensing response. However, because of the rough surface topography of the IDEs, the 3D network exhibits a non-close-packed configuration. This structural characteristic results in a reduced filling factor, which in turn constrains the maximum achievable surface area per unit footprint of the DMC-sensitive films.

## 3. Experiment

### 3.1. Experimental Setup for Humidity Sensing Measurement

**Figure 4:**
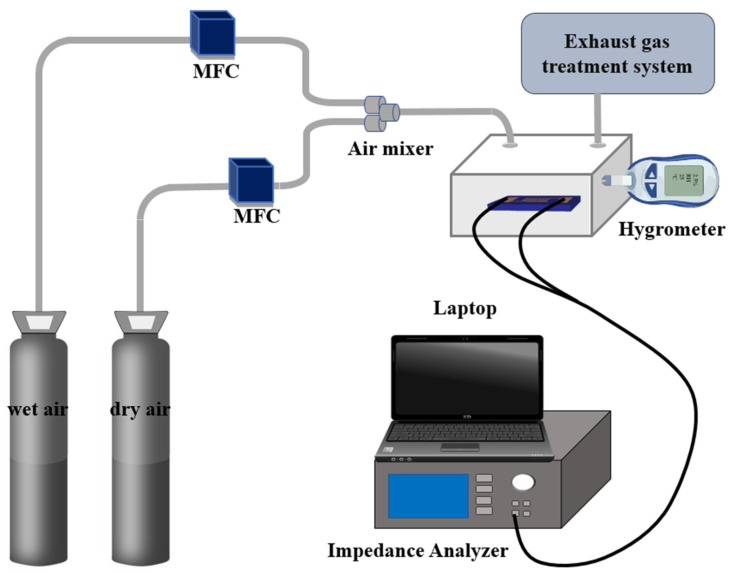
Experimental setup of relative humidity sensing measurement.

Figure 4 schematically illustrates the experimental setup for measuring the RH sensing performance of the sensor. Two mass flow controllers (MFCs; Beijing Qixing Co., Ltd., Beijing, China) independently regulated the flow velocities of dry and wet air streams. The humidity of the mixed airflow was regulated by independently adjusting the flow rates of the dry and wet air streams, while the ambient temperature was maintained at 26 ± 1 °C using an air conditioner. The fabricated sample was placed in a test chamber, with gold electrodes connected to its interdigital electrodes. The sample’s impedance modulus was measured under an applied AC voltage of 1 V at 100 Hz. Variations in impedance modulus were recorded using an impedance analyzer (UCE UC8002, Changzhou Youce Electronic Technology Co., Ltd., Changzhou, China), with data acquisition and storage performed through real-time computer interfacing. A commercial thermohygrometer (Testo model 175H1, Testo SE & Co. KGaA, Titisee-Neustadt, Germany) was employed to track the RH and temperature of the sensor throughout the experiment.

For measuring the dynamic response of the sensor, the humidity of the chamber was changed between high humidity (16% RH) and low humidity (1.1% RH) periodically controlled by the two MFCs.

### 3.2. Performance of the Humidity Sensor

**Figure 5:**
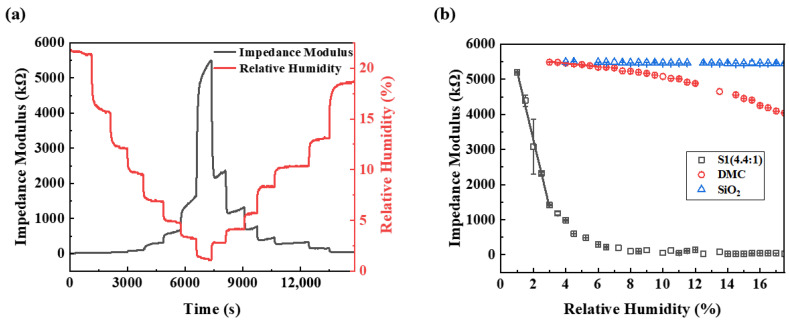
(**a**) Variations in relative humidity and the impedance modulus of 3D DMC network sensor S1 (DMC-to-SiO_2_ mass feed ratio 4.4:1); (**b**) humidity sensing performance of S1, pure DMC, and pure SiO_2_ microspheres.

The RH inside the chamber was decreased from 21.5% RH to 1.1% RH and then increased back to 18.6% RH with a step of approximately 2% RH. The temperature of the chamber was maintained at 26 ± 1 °C. It took 15 min for each step (3 min for the humidity transition and 12 min under constant RH conditions). Figure 5 depicts the variations in relative humidity in the test chamber and the impedance modulus of the fabricated 3D DMC structure sensor (S1, DMC-to-SiO_2_ mass feed ratio is 4.4:1). As demonstrated in Figure 5a, the impedance modulus of the S1 sensor closely tracks humidity changes in real time with synchronous responsiveness. At a relative humidity of 21.5% RH, the measured impedance modulus was 16.22 kΩ. When the relative humidity decreased from 21.5% RH to 1.1% RH, the impedance modulus increased by approximately 5482.65 kΩ (from 16.22 to 5498.87 kΩ). Conversely, when the relative humidity subsequently increased from 1.1% RH to 18.6% RH, the impedance decreased by approximately 5458.27 kΩ (from 5498.87 to 40.60 kΩ). The sensor achieved a 13,544% relative impedance variation over the 1–18.6% RH range, and this represents the highest response magnitude among previously reported humidity sensors utilizing polyelectrolytes.

To evaluate the humidity sensing enhancement of the 3D DMC network structure, comparative tests were performed on parallel sensors made of pure DMC film, pure SiO_2_ microspheres, and the 3D DMC network structure (S1). Figure 5b shows the impedance modulus vs. humidity relationships of these parallel sensors. As evidenced in Figure 5b, the sensor composed of SiO_2_ microspheres without DMC film exhibits a negligible response to low humidity (denoted by blue triangles). The sensor composed of pure DMC film shows a humidity sensitivity of −46.97 kΩ/%RH in the 2–6% RH range and −83.31 kΩ/%RH in the 7–12% RH range (denoted by red circles). The black rectangle denotes the humidity sensing performance of S1. Although the overall relationship between S1’s impedance modulus and humidity is nonlinear, a linear response region is discernible at low humidity levels (1% RH to 3% RH). Linear fitting yields a humidity sensitivity of −1927.68 kΩ/%RH for the 3D DMC sensor (S1), with a high linear coefficient of 0.993. The sensitivity of the 3D DMC sensor is twenty times higher than that of the pure DMC film sensor. The 3D network structure formed by the self-assembled packing of DMC/SiO_2_ microspheres not only increases the surface area of humidity-sensitive films per unit footprint while avoiding invalid stacking, but also ensures uniform and efficient interaction between water molecules and the humidity-sensitive films through its inherent channels, thus significantly enhancing the humidity sensor’s sensitivity.

**Figure 6:**
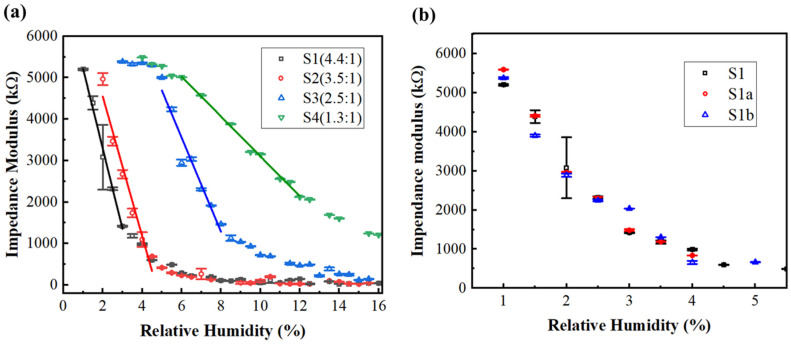
(**a**) Humidity sensing performance of DMC/SiO_2_ composite microsphere sensors (different DMC-to-SiO_2_ mass feed ratios of 4.4:1, 3.5:1, 2.5:1 and 1.3:1). (**b**) Impedance modulus as a function of relative humidity for three parallel sensors (S1, S1a, S1b) fabricated under identical conditions (mass feed ratio 4.4:1, same drop-casting volume).

To systematically investigate the correlation between humidity sensing performance and mass ratio in the DMC/SiO_2_ composite microspheres, samples were prepared at four DMC-to-SiO_2_ mass ratios (4.4:1, 3.5:1, 2.5:1, and 1.3:1), denoted as S1, S2, S3, and S4, respectively. The relationship between impedance modulus and relative humidity of the resulting sensors is presented in Figure 6a (black rectangle for S1, red circle for S2, blue triangle for S3, and green triangle for S4). It is evident that each sample has a distinct linear humidity region, the linear fitting lines are shown in Figure 6a (black line for S1, red line for S2, blue line for S3, and green line for S4) and the fitting results of these four samples with different mass feed ratio are listed in Table 1. These four samples exhibit high sensitivity and linearity within the low humidity range (below 10% RH). The linear response segment identified for our sensors provides a reliable calibration zone and a sensitive probe for the material’s interaction with water molecules at ultra-low concentrations, which is essential for the trace moisture monitoring. The results of S1, S2, S3, and S4 indicate that the humidity sensitivity increases with a rising DMC mass percentage. This is attributed to the increased thickness of the DMC film coated around SiO_2_ microspheres, which intensifies the interactions between DMC and water molecules. Furthermore, as the DMC-to-SiO_2_ mass feed ratio increases, the humidity detection window of the sensor shifts toward the lower humidity range. This is attributed to the thicker DMC film enhancing the absorption of water molecules and lowering the humidity threshold required for sensor saturation.

Two sensors samples were prepared at the same condition of S1 (mass feed ratio 4.4:1), denoted as S1a and S1b, respectively. The relationship between impedance modulus and relative humidity of the parallel sensors is presented in Figure 6b (black rectangle for S1, red circle for S1a, blue triangle for S1b). The impedance data points of the three parallel sensors with respect to relative humidity roughly coincide. Their humidity-sensitive region is consistently located within 1–3% RH. This demonstrates the reproducibility of our sensor fabrication process.

Humidity hysteresis is one of the most important characteristics for evaluating the performance of humidity sensors. In our experiment, the humidity response of the sensors was measured from 1% to 20% RH during the adsorption process and in the reverse direction for the desorption process. Humidity hysteresis is defined as the maximum RH difference at the same impedance modulus between the adsorption and desorption curves of the sensor [26]. As shown in Table 1, the S1 sample exhibits a relatively large hysteresis of 1.4% RH at approximately 2% RH, which corresponds to the large error observed in Figure 6a. This behavior can be attributed to the large hysteresis and high sensitivity of S1 at this humidity point, as the measurements were performed over a broad humidity range (1–20% RH). To validate this inference, an additional hysteresis cycling test was conducted over a narrower humidity range (1–5% RH) using a parallel sample (S1a). The measured hysteresis value was substantially reduced to 0.48% RH within this narrow range (1–5% RH). This result indicates that the larger hysteresis observed previously over the wider cycling range (1–20% RH) can be partly ascribed to the cumulative effect of water adsorption/desorption kinetics within the sensing material across a broad humidity interval. As shown in Figure 6b, when the sensors (S1a, S1b) operate within the low-humidity range (1–5% RH), the measurement error is relatively small.

**Figure 7:**
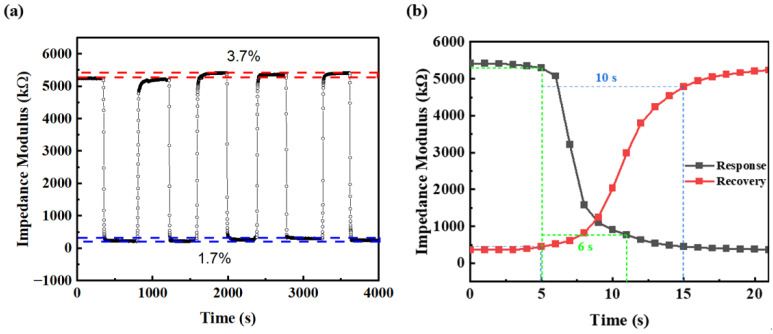
(**a**) Transient response curve of S1 humidity sensor between 1% RH and 16% RH for five cycles; (**b**) response and recovery measurement of S1 sensor. The green dashed line represents the response time of 6 s, and the blue dashed line represents the recovery time of 10 s.

**Figure 8:**
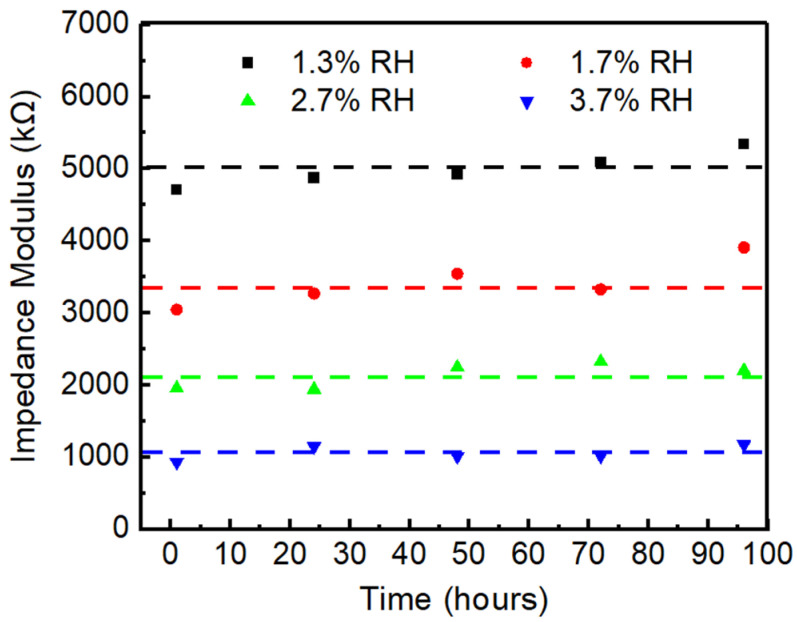
Long-term stability of the S1a sensor within 96 h.

The response and recovery time is one of the most important parameters for humidity sensors. In this work, the response and recovery times are defined as the time required for the impedance modulus to reach 90% of the total change during adsorption and desorption processes, respectively. As shown in Figure 7b, the response and recovery times were determined as 6 s and 10 s, respectively. The rapid response and recovery can be attributed to the interconnected channels formed by particle stacking within the sensing film, which facilitate the rapid transport of water molecules. Figure 7a shows the repeatability and stability characteristics of the DMC/SiO_2_ sensor (S1). During the humidity change between 1.1% RH and 16% RH, the calculated relative standard deviation (RSD) of the sensor’s total impedance variation amplitude (between 1.1% RH and 16% RH) across 5 consecutive cyclic tests was 1.453%, demonstrating excellent repeatability. Furthermore, the baseline drift values corresponding to 1.1% RH and 16% RH accounted for 3.7% and 1.7% of the total impedance variation amplitude, respectively, indicating good repeatability of the S1 sensor under dynamic conditions. Moreover, the impedance modulus of the S1a sensor at different low humidities exhibited negligible variation over a 96 h period (Figure 8), demonstrating its excellent long-term stability.

To verify the accuracy of the 3D DMC humidity sensor (S1) for RH detection, cross-sensitivity tests were performed using acetone (C_3_H_6_O) and ethanol (C_2_H_6_O). All experiments were carried out at 3.2% RH and 25 °C. The sensor responses to 165 ppm acetone and 208 ppm ethanol were 11.05 kΩ and 14.47 kΩ, respectively. Its responses to the two volatile organic gases can be considered negligible.

**Figure 9:**
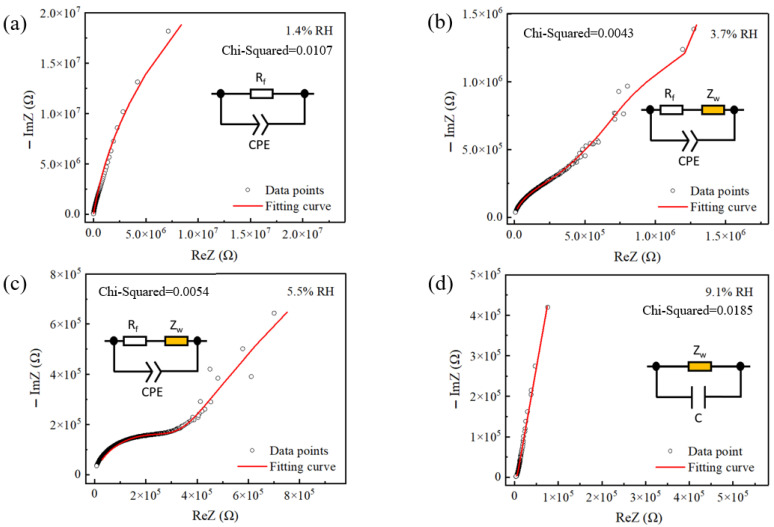
Complex impedance spectra and corresponding equivalent circuits of the S2 sensor at (**a**) 1.4% RH, (**b**) 3.7% RH, (**c**) 5.5% RH and (**d**) 9.1% RH.

In order to investigate the humidity sensing mechanism, the complex impedance spectra (CIS) of the S1 sensor were measured at different humidities over the operating frequency range of 20 Hz to 20 kHz. Figure 9 shows the CIS and equivalent circuit (EC) of the 3D DMC humidity sensor under the conditions of different RH levels. Based on our experimental findings and the relevant literature, we put forward a reasonable hypothetical mechanism to explain the observed sensing behavior. At 1.4% RH, the CIS curve presents a high-curvature semicircle characteristic of predominantly capacitive behavior. Charge transport relies on charging/discharging due to the lack of conductive pathways [27]. The data were fitted to a constant phase element (CPE) parallel with sensing film bulk resistance (R_f_), whose high value (~78.5 MΩ) confirms the film’s insulating state under dry conditions.

As humidity increases to 3.7% RH and 5.5% RH, shown in Figure 9b,c, the CIS evolves into a semicircle followed by a straight line. Concomitantly, the semicircle radius decreases, and the data are fitted to an equivalent circuit comprising a resistor (R_f_) in series with Warburg impedance (Z_W_), which is then parallel with a CPE. The decreasing radius corresponds to a dramatic reduction in the fitted R_f_ values (to ~802 kΩ and ~353 kΩ, respectively), which are about two orders of magnitude compared with the 1.4% RH case. This confirms the formation of conductive pathways within the DMC film network. Moreover, the emergence of the Warburg element (W-R, ~6.5 MΩ and ~2.8 MΩ, respectively) indicates that low-frequency transport is already limited by ion diffusion [28]. As reported in similar ionic systems [29], DMC releases mobile Cl^−^ ions even under trace water conditions. We propose that these Cl^−^ ions serve as the primary charge carriers, initiating the conductive path. Concurrently, the adsorbed water molecules facilitate proton hopping via a Grotthuss mechanism along the discontinuous water layer and surface hydroxyl groups [30].

Furthermore, as humidity increases to 9.1% RH, the effect of Warburg impedance grows more pronounced. As shown in Figure 9d, the semicircle almost disappears, and the straight line dominates the CIS at 9.1% RH. The equivalent circuit was simplified to an ideal capacitor in parallel with a Warburg diffusion impedance, demonstrating that the two processes, rapid interfacial charging/discharging and slow ion diffusion, can be decoupled. Meanwhile, the Warburg resistance (W-R) drops drastically to 1.90 × 10^4^ Ω, indicating the formation of highly interconnected ion diffusion pathways. This could be ascribed to the formation of a continuous water layer via multilayer physisorption, the DMC sensing film dissociates to release large amounts of Cl^−^ ions, which play a critical role in the conduction of the film [31].

Compared to the single DMC film sensor, the sensitivity of the 3D DMC sensor is enhanced twenty times. In the 3D DMC sensor, the DMC shell layers establish a continuous percolative resistive network across stacked microspheres, while the porous 3D architecture offers a substantially larger accessible interface than planar thin films. In addition, the large pores (hundreds of nanometers) facilitate rapid ingress and egress of water molecules. Although this network is nearly insulating at low RH, a slight RH increase quickly extends adsorbed moisture throughout the interconnected DMC surfaces, activates ionic transport, and markedly lowers resistance across the full 3D film rather than only the top surface. As a result, R_f_ drops by nearly two orders of magnitude at low humidity (3.7% RH), producing a large electrical output from a small RH increment. Meanwhile, the emergence and reduction in the Warburg term (W-R) indicate that the same 3D network also shortens diffusion pathways at low frequency, reinforcing the resistance change. Therefore, the key sensitivity gain is not merely higher water uptake, but the 3D DMC resistive network’s ability to convert early-stage adsorption into a film-wide, strongly amplified decrease in effective resistance.

In the study, CIS was performed at open-circuit potential (OCP) to evaluate humidity sensing under bias-free, near-equilibrium conditions. In this redox-probe-free 3D DMC system, OCP minimizes field-induced perturbation and provides a consistent baseline for comparing RH-dependent impedance and device-to-device reproducibility, and was therefore used as the primary testing condition. In the low-humidity region, the decrease in R_f_ is markedly larger than that of the W-R component, suggesting that the response is mainly governed by changes in the bulk conductive network. This trend indicates scope for further optimization via interfacial engineering [27]. In particular, the effects of electrode architecture and applied bias on signal amplitude, noise, and detection limit warrant systematic investigation without altering the dominant sensing pathway.

Table 2 compares the performance of reported low-humidity sensors based on different sensitive materials (including polymers, metal halides, two-dimensional nanomaterials, and their composites). Compared with previous sensors, our 3D DMC network sensor exhibits the highest humidity sensitivity among polyelectrolytic systems. It achieves a 13,544% impedance variation across 1–18.6% RH, which is approximately 5.7 times greater than reported PILs and SiO_2_/PILs sensors [17,23]. Within the ultra-low humidity range (1–3% RH), it attains a sensitivity of −1927.68 kΩ/%RH, which is 3.6 times higher than that of NiI_2_-based sensors [18]. Although its sensitivity is lower than that of the NiBr_2_-based sensor (40 MΩ/%RH) [19], the latter exhibits a negligible response below 7% RH. Moreover, our sensor maintains rapid response and recovery times of 6 s and 10 s, respectively. Its recovery time is sevenfold faster than that of NiBr_2_ variants.

## 4. Conclusions

In summary, an ultra-high sensitivity RH sensor based on 3D DMC network structure has been successfully synthesized as an advanced humidity-sensing platform. The optimized porous architecture of the microspheres synergizes with the hydrophilic groups of DMC, which is beneficial for enhancing the interfacial contact between DMC and water molecules. Concurrently, the inter-spheres channels formed by the stacking of DMC-coated SiO_2_ microspheres enable stable water adsorption in the ultra-low relative humidity range of 1–18.6% RH, while facilitating rapid adsorption and desorption processes across the sensitive layer. The sensor showed an ultrahigh sensitivity of −1927.68 kΩ/%RH within the 1–3% RH range. Meanwhile, the sensor achieves rapid response/recovery times of 6 s/10 s, along with a minimal hysteresis of 1.4% RH. The superior comprehensive performance of the 3D DMC network based RH sensor allows it to possess great potential for application in widespread fields, such as those of chemical processing, medical diagnostics, electricity, and aerospace.

## Figures and Tables

**Table 1:** Linear fitting results of four samples with different mass feed ratios.

Sample	Mass Feed Ratios	Sensitivity (kΩ/% RH)	Linear Coefficient	Resolution (% RH)	Hysteresis
S1	4.4:1	−1927.68 (1–3% RH)	0.993	0.20	1.4% RH
S2	3.5:1	−1682.44 (2–4.5% RH)	0.963	0.15	0.2% RH
S3	2.5:1	−1135.20 (5–8% RH)	0.945	0.09	0.5% RH
S4	1.3:1	−480.55 (6–12% RH)	0.995	0.05	1.4% RH

**Table 2:** Performances of reported low humidity sensors based on different sensitive materials.

Sensitive Material	Humidity Range	Response	Response/Recovery Time	Hysteresis	Reference
PILs	5–35% RH	4800%	1 s/10 s	0.2% RH	[17]
NiI_2_	0.4–11% RH	529 kΩ/%RH	-	-	[18]
NiBr_2_	7–24% RH	40 MΩ/%RH	2 s/71 s	-	[19]
BP	<45% RH	0.2 pF/%RH	3.9 s/1 s	3% RH	[16]
SnO_2_/MoS_2_	0–20% RH	387.5 μF/%RH	15 s/40 s	-	[20]
PPy/Ag/TiO_2_ NPs	173.9–9711 ppmv	0.0069 Hz/ppmv	12 s/-	-	[21]
QC-P4VP/PANI	1–15% RH	860%	24 s/35 s	3% RH	[32]
QC-P4VP/RGO	0.18–2.1% RH	500%	21 s/78 s	4.5% RH	[22]
SiO_2_@PILs	7–33% RH	4112%	3 s/259 s	0.6% RH	[23]
SiO_2_@PILs	7–33% RH	806%	8 s/13 s	1% RH	[24]
ILs/MOF-AA	5–30% RH	88%	0.6 s/1.7 s	0.2% RH	[25]
DMC/SiO_2_	1–18.6% RH 1–3% RH 2–4.5% RH	S1: 13,544% S1: −1927.68 kΩ/%RH S2: −1682.44 kΩ/%RH	6 s/10 s	1.4% RH 0.2% RH	This work

## Data Availability

The data presented in this study are available on request from the corresponding author. The data are not publicly available due to ongoing study.

## Abbreviations

The following abbreviations are used in this manuscript:

RH	Relative humidity
DMC	Methacryloxyethyl trimethyl ammonium chloride
3D	Three-dimensional

## References

[1] Kok S.P., Go Y.I., Wong M.L. (2025). Effect of Hygroscopic and Electronic Materials, Challenges, and Sensing Requirements in Humidity-Controlled Industry. J. Electron. Mater..

[2] Ku C.A., Chung C.K. (2023). Advances in humidity nanosensors and their application. Sensors.

[3] Prasetya N., Okur S. (2024). Investigation of the free-base Zr-porphyrin MOFs as relative humidity sensors for an indoor setting. Sens. Actuators A Phys..

[4] Feng D., Zheng H., Sun H., Li J., Xi J., Deng L., Guo Y., Jiang B., Zhao J. (2023). SnO_2_/polyvinyl alcohol nanofibers wrapped tilted fiber grating for high-sensitive humidity sensing and fast human breath monitoring. Sens. Actuators B Chem..

[5] Yap K.Z., Lim W.Y., Kalkal A., Gopinath P., Ramakrishnan N. (2023). Electrophoretic deposited Mg ion functionalized graphene oxide-based ultrahighly sensitive humidity sensor. IEEE Trans. Instrum. Meas..

[6] Wu J., Lu P., Dai J., Zheng C., Zhang T., Yu W.W., Zhang Y. (2021). High performance humidity sensing property of Ti_3_C_2_T_x_ MXene-derived Ti_3_C_2_T_x_/K_2_Ti_4_O_9_ composites. Sens. Actuators B Chem..

[7] Qi R., Zhang T., Guan X., Dai J., Liu S., Zhao H., Fei T. (2020). Capacitive humidity sensors based on mesoporous silica and poly (3, 4-ethylenedioxythiophene) composites. J. Colloid Interface Sci..

[8] Yang C., Gu W., Yu S., Zhang H. (2021). Effect of surfactant on performance of ZnO humidity sensor. Optoelectron. Lett..

[9] Guan X., Hou Z., Wu K., Zhao H., Liu S., Fei T., Zhang T. (2021). Flexible humidity sensor based on modified cellulose paper. Sens. Actuators B Chem..

[10] Ma Z., Fei T., Zhang T. (2023). An overview: Sensors for low humidity detection. Sens. Actuators B Chem..

[11] Kumar L., Islam T., Mukhopadhyay S.C. (2017). Sensitivity enhancement of a PPM level capacitive moisture sensor. Electronics.

[12] Pal S., Das A., Nandy S., Kar R., Ghosh J. (2019). Development of a near-infrared tunable diode laser absorption spectrometer for trace moisture measurements in helium gas. Rev. Sci. Instrum..

[13] Han X., Xia S., Cao J., Wang C., Chen M. (2021). Effect of Humidity on Properties of Lithium-ion Batteries. Int. J. Electrochem. Sci..

[14] Lim K.-H., Huh J.-E., Lee J., Cho N.-K., Park J., Nam B., Lee E., Kim Y.S. (2017). Strong Influence of Humidity on Low-Temperature Thin-Film Fabrication via Metal Aqua Complex for High Performance Oxide Semiconductor Thin-Film Transistors. ACS Appl. Mater. Interfaces.

[15] Zargar Z.H., Akram K.J., Biswal G.R., Islam T. (2021). A Linear Capacitive Sensor for ppm Moisture Measurement in SF_6_ Gas-Insulated Switchgear. IEEE Trans. Instrum. Meas..

[16] Chen W., Huang J., Zhu C., Xu F., Huang Q.A. (2019). An interdigital capacitive humidity sensor with layered black phosphorus flakes as a sensing material. IEEE Sens. J..

[17] Yu Y., Ma Z., Miao X., Cui Y., Song Y., Liu S., Fei T., Zhang T. (2024). Humidity sensors based on cross-linked poly (ionic liquid) s for low humidity sensing. Sens. Actuators B Chem..

[18] Zhang Y., Ren J., Wu Y., Zhong X., Luo T., Cao J., Yin M., Huang M., Zhang Z. (2020). Application of moisture-induced discoloration material nickel (II) iodide in humidity detection. Sens. Actuators B Chem..

[19] Yin M., Hu J., Huang M., Chen P., Zhang Y. (2021). Moisture-induced reversible material transition behavior of nickel (II) bromide for low humidity detection. Sens. Actuators A Phys..

[20] Zhao Y., Yang B., Liu J., Chen X., Wang X., Yang C. An ultrasensitive humidity sensor based on SnO_2_-modified MoS_2_ nanocomposite at low-humidity range. Proceedings of the 2018 IEEE Micro Electro Mechanical Systems (MEMS).

[21] Su P.G., Chang Y.P. (2008). Low-humidity sensor based on a quartz-crystal microbalance coated with polypyrrole/Ag/TiO_2_ nanoparticles composite thin films. Sens. Actuators B Chem..

[22] Li Y., Fan K., Ban H., Yang M. (2016). Detection of very low humidity using polyelectrolyte/graphene bilayer humidity sensors. Sens. Actuators B Chem..

[23] Ma Z., Miao X., Song Y., Yu Y., Wu K., Liu S., Fei T., Zhang T. (2023). Construction of Dual—Channel Water Transport in Mesoporous Silica Low Humidity Sensors to Achieve High Sensitivity. Small.

[24] Ma Z., Song Y., Zhao H., Liu S., Yang X., Fei T., Zhang T. (2024). Mesoporous Silica Modified by Poly (Ionic Liquid) s for Low-Humidity Sensing. IEEE Sens. J..

[25] Wu K., Yu Y., Hou Z., Guan X., Zhao H., Liu S., Yang X., Fei T., Zhang T. (2022). A humidity sensor based on ionic liquid modified metal organic frameworks for low humidity detection. Sens. Actuators B Chem..

[26] Dong W., Ma Z., Duan Q. (2018). Preparation of stable crosslinked polyelectrolyte and the application for humidity sensing. Sens. Actuators B Chem..

[27] Randviir E.P., Banks C.E. (2022). A review of electrochemical impedance spectroscopy for bioanalytical sensors. Anal. Methods.

[28] Yeh Y.C., Tseng T.Y. (1989). Analysis of the dc and ac properties of K_2_O-doped porous Ba_0.5_Sr_0.5_TiO_3_ ceramic humidity sensor. J. Mater. Sci..

[29] Park Y.D., Kang B., Lim H.S., Cho K., Kang M.S., Cho J.H. (2013). Polyelectrolyte Interlayer for Ultra-Sensitive Organic Transistor Humidity Sensors. ACS Appl. Mater. Interfaces.

[30] Ernsberger F.M. (1983). The nonconformist ion. J. Am. Ceram. Soc..

[31] Dai J., Zhang T., Zhao H., Fei T. (2017). Preparation of organic-inorganic hybrid polymers and their humidity sensing properties. Sens. Actuators B Chem..

[32] Li Y., Fan K., Ban H., Yang M. (2015). Bilayer-structured composite sensor based on polyaniline and polyelectrolyte for sensitive detection of low humidity. Synth. Met..

